# Immune System and Hepatic Stellate Cells’ Crosstalk in Liver Fibrosis: Pathways and Therapeutic Potential

**DOI:** 10.1155/jimr/2656395

**Published:** 2026-01-02

**Authors:** Wahyu Widowati, Adilah Hafizha Nur Sabrina, Annisa Firdaus Sutendi, Fadhilah Haifa Zahiroh, Aris Muhamad Nurjamil, Teresa Liliana Wargasetia, Ita Margaretha Nainggolan, Rizal Azis, Elham Rismani, Massoud Vosough

**Affiliations:** ^1^ Faculty of Medicine, Maranatha Christian University, Bandung, West Java, 40164, Indonesia; ^2^ Aretha Medika Utama, Biomolecular and Biomedical Research Center, Bandung, 40163, West Java, Indonesia; ^3^ School of Medicine and Health Sciences, Atma Jaya Catholic University of Indonesia, Jakarta, 14440, Indonesia, atmajaya.ac.id; ^4^ Biomedical Engineering, Department of Electrical Engineering, Faculty of Engineering, University of Indonesia, Depok, Indonesia, ui.ac.id; ^5^ Molecular Medicine Department, Biotechnology Research Center (BRC), Pasteur Institute of Iran, Tehran, Iran, pasteur.ac.ir; ^6^ Department of Regenerative Medicine, Cell Science Research Center, Royan Institute for Stem Cell Biology and Technology, Tehran, Iran, royaninstitute.org; ^7^ Experimental Cancer Medicine, Institution for Laboratory Medicine, and Karolinska University Hospital, Karolinska Institute, Stockholm, Sweden, ki.se; ^8^ Department of Cellular and Molecular Biology, Faculty of Sciences and Advanced Technology in Biology, University of Science and Culture, Tehran, Iran, usc.ac.ir

**Keywords:** extracellular matrix (ECM), hepatic stellate cells (HSCs), immune system, liver fibrosis, therapeutic strategies

## Abstract

Liver fibrosis, characterized by the excessive deposition of extracellular matrix (ECM) driven by hepatic stellate cells (HSCs) activation, remains a critical challenge due to its progression to cirrhosis and hepatocellular carcinoma (HCC). This review clarifies the complex crosstalk between the immune system and HSCs, highlighting key cellular players including macrophages, natural killer (NK) cells, regulatory T cells (Tregs), and their cytokine‐mediated signaling pathways that regulate fibrogenesis and fibrosis resolution. We describe pivotal molecular mechanisms such as transforming growth factor (TGF)‐β, platelet‐derived growth factor (PDGF), Wnt/β‐catenin, and NF‐κB signaling in HSCs modulation, emphasizing their interplay with immune responses. Novel therapeutic strategies targeting this complex immune–HSCs interaction, ranging from immunomodulatory agents, macrophage polarization, and NK cell‐based therapies, to stem cell‐derived exosomes, offer promising opportunities for preventing and reversing fibrosis. We further discuss innovative combination therapies integrating immunotherapies with antifibrotic agents, personalized strategies based on immune profiling, and the challenges of immune heterogeneity in fibrosis management. This review discusses recent advances in molecular interplay of immune system and HSCs, highlighting novel therapeutic targets, and future perspectives for managing chronic liver diseases.

## 1. Introduction

Liver fibrosis is defined as the excessive deposition of extracellular matrix (ECM) components in the liver, resulting from chronic liver injury and inflammation. This pathological process is characterized by the modulation of hepatic stellate cells (HSCs), which play a crucial role in the fibrogenesis process by producing collagen and other ECM proteins in response to hepatocyte damage and inflammatory stimuli [1–3]. Liver fibrosis is significant due to its ability to advance into serious liver disorders, such as cirrhosis, liver failure, and hepatocellular carcinoma (HCC), thus playing a major role in the morbidity and mortality associated with liver diseases [1, 4]. Recent experimental evidence further supports the direct role of fibrotic ECM in promoting cancerous phenotypes in HCC models, emphasizing the contribution of the fibrotic microenvironment to tumor progression [5].

The pathophysiology of liver fibrosis is multifactorial, often triggered by various etiologies such as viral hepatitis, alcoholic liver disease, and metabolic dysfunction‐associated steatotic liver disease (MASLD) (formerly NAFLD) [4]. In response to injury, the liver undergoes an intricate interaction of cellular processes, where immune cell activation leads to the release of proinflammatory cytokines, intensifying liver damage [6]. Moreover, liver fibrosis is not merely a passive scarring process; it is a dynamic and potentially reversible condition, particularly in its early stages [7]. If the underlying cause of liver injury is addressed, such as through antiviral therapy for hepatitis or lifestyle changes for MASLD, there is potential for regression of fibrosis [2, 7]. However, once fibrosis progresses to cirrhosis, the damage becomes largely irreversible, underscoring the importance of early detection and intervention.

Despite significant advancements, current therapies often target underlying causes of liver injury rather than directly addressing fibrosis. Immunomodulatory and antifibrotic approaches show promise but face challenges related to heterogeneity in immune responses and fibrosis stages [8]. This underscores the urgent need for innovative therapies that target the immune microenvironment and HSCs modulation simultaneously.

Recent studies have revealed the pivotal role of immune system and HSCs crosstalk in fibrosis progression and resolution. Macrophages, natural killer (NK) cells [9], and regulatory T cells (Tregs) facilitate these interactions via cytokines and signaling pathways, as well as through exosomal communication [10]. Emerging therapeutic strategies, including NK cell‐driven therapies, macrophage polarization, and exosome‐based interventions, exploit these interactions to modulate fibrosis [11]. The objective of this review is to explain these interactions and their contributions to the dysregulated accumulation of ECM components, a hallmark of fibrotic progression. By characterizing the signaling pathways and modulatory factors involved in this immune system and HSCs interplay, we aim to uncover novel therapeutic targets with the potential to attenuate or reverse fibrotic features, providing a foundation for designing advanced therapeutic modalities in chronic liver diseases.

## 2. Pathophysiology of Liver Fibrosis

### 2.1. Role of HSCs in Liver Fibrosis

Following liver injury, quiescent HSCs undergo activation and transdifferentiate into myofibroblast‐like cells, a process critical for wound healing. Activated HSCs proliferate and secrete ECM components, including collagen and fibronectin, in response to inflammatory mediators released by damaged hepatocytes and liver cells, such as Kupffer cells [12]. Activated HSCs express α‐SMA, a marker of their transdifferentiated state, which correlates with increased ECM deposition and fibrotic scar formation [13]. The accumulation of ECM, coupled with decreased matrix metalloproteinases (MMPs) activity, fosters fibrosis progression, perpetuating HSCs activity, and the fibrotic response [14, 15].

HSC activation is largely influenced by a combination of cytokines, growth factors, and oxidative stress. In addition, several signaling pathways such as platelet‐derived growth factor (PDGF), Wnt/β‐catenin, and nuclear factor‐kappa B (NF‐κB) also play significant roles in HSC modulation [16, 17]. Transforming growth factor (TGF)‐β is a key fibrogenic cytokine that promotes HSCs activation and ECM production. TGF‐β primarily activates HSCs through a Smad‐dependent mechanism; when TGF‐β binds to its receptor, Smad2 and Smad3 are phosphorylated and translocated into the nucleus, where they regulate fibrogenic gene expression [18]. TGF‐β initiates the expression of α‐SMA and other myofibroblast markers, facilitating HSCs differentiation [19]. Notably, recent mechanistic studies indicate that hydronidone ameliorate liver fibrosis by inhibiting HSCs activation through a mechanism involving Smad7‐mediated degradation of TGF‐β receptor I (TGFβRI). Knockdown of Smad7 in HSCs reversed the protective effects, confirming Smad7’s central role in the antifibrotic mechanism [20]. This dynamic regulation highlights a mechanistic checkpoint that could be leveraged for therapeutic purposes. Furthermore, TGF‐β activates noncanonical pathways, including MAPK and PI3K/Akt, which further contribute to HSCs modulation and ECM production. Additionally, Solhi et al. [21] demonstrated that TGF‐β treatment of human LX‐2 cells induces activation markers and triggers fibrosis‐related signaling pathways including hedgehog signaling and endoplasmic reticulum stress, further supporting the pivotal role of TGF‐β in HSCs activation and fibrogenesis. These mechanistic insights highlight how TGF‐β signaling complexity impacts fibrosis progression and suggest that targeted modulation of Smad7 and noncanonical pathways could enhance antifibrotic strategies.

The PDGF signaling pathway is another key regulator of HSCs activation, which primarily promotes HSCs proliferation and migration. PDGF, which is increased in response to liver injury, binds to its receptor on HSCs, activating downstream pathways, such as PI3K and RhoA/ROCK, which increase ECM production and promote a myofibroblast‐like phenotype in HSCs. This pathway accelerates the fibrotic process by increasing the number of activated HSCs that produce ECM. PDGF receptors (PDGFRs), especially PDGFR‐α and PDGFR‐β, have key roles in the functional differentiation of HSCs during the process of liver fibrosis. Among them, PDGFR‐β is of greater significance as it shows the highest affinity to PDGF‐BB, a potent mitogen for HSCs [22].

Activation of PDGFR‐β triggers proliferation, migration, and trans‐differentiation of HSCs into myofibroblasts, which are the main components in the fibrogenesis process [23]. Activation of these receptors initiates downstream signaling cascades, including the Ras/Raf/MEK/ERK and PI3K/Akt pathways, which play an important role in cell survival and fibrotic tissue formation [24]. In chronic liver injury, PDGFR‐β expression is often identified as a key marker of HSCs activation, with a particularly significant role in profibrogenic effects [25]. Experimental models indicate that PDGFR‐β antagonists can reduce fibrosis by limiting the activated HSC population, emphasizing the receptor’s therapeutic relevance [26]. In contrast, PDGFR‐α also contributes to HSCs activation, although its role is considered less dominant compared to PDGFR‐β [27]. However, differential roles of PDGFR‐α and PDGFR‐β suggest a more sophisticated therapeutic targeting, given that PDGFR‐α may contribute less dominantly but distinctly in the remodeling of fibrotic tissue [27]. Pharmacological blockade of PDGFR‐β signaling has effectively attenuated fibrosis in preclinical models, which supports ongoing efforts to refine targeted anti‐PDGF approaches. Kocabayoglu et al. [28] demonstrated that deletion of β‐PDGFR in HSCs attenuates liver fibrosis in vivo in mouse models induced by carbon tetrachloride (CCl4) and bile duct ligation (BDL). Additionally, destruxin A5, a compound inhibiting PDGF‐BB/PDGFR‐β signaling, alleviated liver fibrosis in BDL‐treated mice, reducing α‐SMA expression and collagen deposition in preclinical studies, further supporting the therapeutic promise of PDGFR‐β blockade [29]. These mechanistic aspects necessitate further investigation to enhance anti‐PDGF strategies.

Wnt/β‐catenin signaling also contributes to HSCs activation. Activation of this pathway stabilizes β‐catenin, which accumulates in the cytoplasm and is subsequently translocated to the nucleus, driving the expression of fibrogenic genes [30]. Chronic liver injury specifically involves this pathway, leading to persistent HSCs activity and the development of fibrosis [31]. This pathway interacts closely with other signaling pathways, such as TGF‐β and NF‐κB, to influence HSCs behavior. TGF‐β, known to be a major trigger of HSCs activation and fibrosis, can be affected by Wnt/β‐catenin signaling, which supports hepatocyte proliferation and tissue repair. The interaction of β‐catenin with Smad proteins, which are downstream effectors of TGF‐β, creates a complex regulatory network that can promote or inhibit fibrogenesis depending on the context [32]. In addition, the NF‐κB pathway, which is often active during inflammatory responses, can modulate Wnt/β‐catenin activity through feedback that modulates HSCs and the overall fibrotic response [33]. As a transcription factor involved in the inflammatory response, NF‐κB is activated in HSCs during liver injury, which induces the production of proinflammatory cytokines and chemokines that enhance HSCs activation and fibrogenesis [34]. This pathway perpetuates liver injury by promoting inflammatory signaling in activated HSCs. Moreover, Sun et al. [35] highlighted the therapeutic potential of modulating NF‐κB and Wnt/β‐catenin signaling pathways via the inhibitory peptide for HSCs activation that contribute to the inhibition of fibrogenic pathways. Inhibition of MAPK and NF‐κB signaling pathways alleviates CCl4‐induced liver fibrosis in Toll‐like receptor 5 (TLR5) deficient mice. TLR5 deficiency reduces MAPK (p38 and ERK) and NF‐κB activation in HSCs, leading to decreased inflammatory cytokines, fibrotic gene expression, collagen deposition, and liver inflammation. This highlights the critical role of TLR5‐mediated MAPK/NF‐κB signaling in HSC activation and liver fibrogenesis [36].

Ferroptosis, as a regulated, iron‐dependent cell death driven by lipid peroxidation, is a pivotal modulator in liver fibrosis and HCC progression [37]. Mechanistically, the susceptibility of HSCs to ferroptosis is regulated by pathways involving ferritinophagy mediated by NCOA4 and the system Xc^−^ component SLC7A11, which conducts cystine uptake and glutathione synthesis. Experimental inhibition of SLC7A11 sensitizes activated HSCs to ferroptosis, resulting in decreased collagen deposition and reversal of fibrosis in murine models. However, genetic or pharmacologic impairment of ferroptosis has been shown to increase hepatocyte survival but may paradoxically promote oncogenic transformation in chronic models, emphasizing that ferroptosis modulation is highly context‐dependent. These findings highlight ferroptosis as a therapeutic target, but also emphasize on the necessity for precision interventions to differentially modulate cell death in fibrogenic versus parenchymal cells [38, 39].

Epigenetic modifications, such as histone changes and noncoding RNA influences, can regulate β‐catenin expression and activity, ultimately affecting HSCs fate decisions. Shi et al. [40] reviewed histone modifications and chromatin remodeling that regulate HSC activation and inflammation, including evidence of enhancer of zeste homolog 2 (EZH2) and histone acetyltransferases modifying fibrogenic gene transcription in HSCs. For example, histone acetyltransferase p300 enhances HSC response to TGF‐β and modulates β‐catenin signaling, while histone deacetylase SIRT1 suppresses HSC trans‐differentiation by counteracting these epigenetic effects, suggesting that epigenetic modifications could be a potential therapeutic target for manipulating Wnt signaling in HSCs [41–43]. Moreover, long noncoding RNAs have been implicated in the regulation of the Wnt/β‐catenin pathway, adding an additional layer of control to HSCs activation and liver fibrosis [44, 45]. A deeper understanding of the interactions between the Wnt/β‐catenin pathway, other signals, such as TGF‐β and NF‐κB, as well as epigenetic factors, is essential for developing therapies that can support liver regeneration while preventing fibrosis.

Mitogenic signaling pathways, including those mediated by HGF, EGF, and PDGF are vital in orchestrating both normal and pathological liver regeneration. Dysregulation of these pathways can shift the balance from effective tissue repair toward aberrant regeneration and fibrosis [46]. Moreover, dysregulated crosstalk among HGF, EGF, PDGF, and inhibitory factors like TGF‐β disrupts the tightly controlled regenerative process. For example, overexpression of PDGF can catalyze pathological trans‐differentiation of HSCs into proliferative myofibroblasts, perpetuating chronic fibrosis [28, 47, 48].

Angiotensin II and ROS are additional contributors to HSCs activation. Angiotensin II stimulates HSCs through the angiotensin II receptor, which promotes fibrogenesis by increasing ECM production and enhancing HSC contractility [49]. ROS, which are produced in response to oxidative stress, have dual roles: low levels of ROS stimulate HSCs activity and proliferation, while excessive ROS can cause cell damage and apoptosis [50]. The NADPH oxidase (NOX) family, particularly NOX1 and NOX4, are the major sources of ROS in HSCs and play a critical role in regulating fibrogenesis [51, 52].

Overall, the complex signaling and ECM production involved in HSCs activation underscore their dual role in liver regeneration and the risk of pathological fibrosis with sustained activation. The balance between HSCs activation and apoptosis is critical; factors such as tissue inhibitors of metalloproteinases (TIMPs) can inhibit HSCs apoptosis, leading to their accumulation and continued fibrogenesis [53]. Thus, although HSCs are essential for liver repair, their dysregulation may contribute to fibrotic disease.

### 2.2. Immune System Involvement in Liver Fibrosis

**Figure 1:**
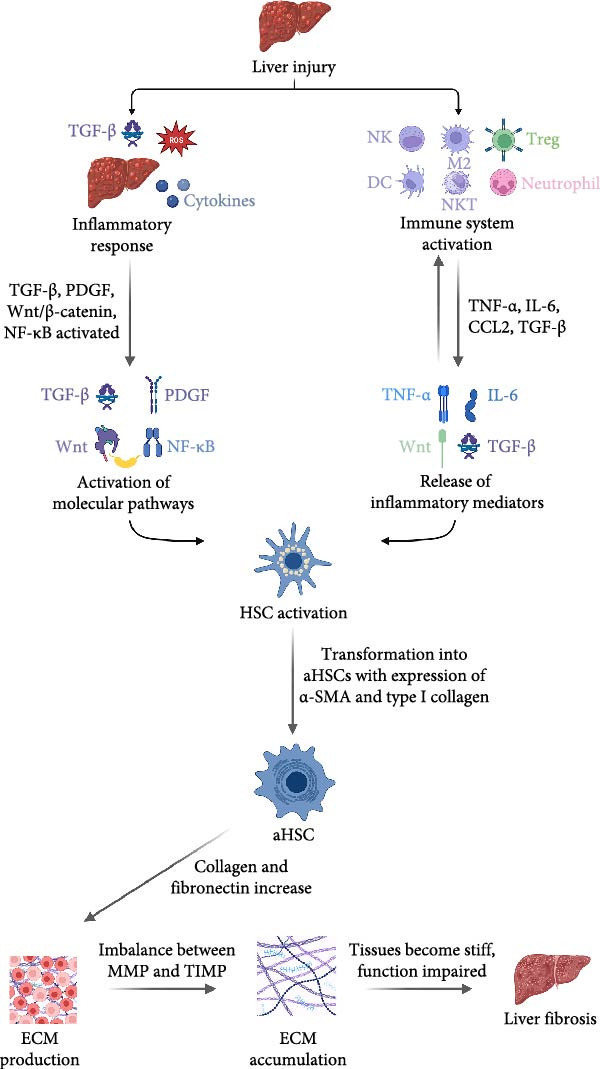
Chronological mechanism of liver fibrosis progression and resolution, illustrating the interplay between liver injury, immune system activation, release of inflammatory mediators, HSCs activation, ECM production, and ECM accumulation.

Liver fibrosis is a complex process influenced by various immune cells and inflammatory mediators (Figure 1). Interactions between immune cells are mediated through cytokine signals and direct cell‐to‐cell contact, which influence the dynamics between inflammation and tissue repair. This relationship demonstrates the complexity of the communication network in the fibrotic microenvironment. The fibrotic microenvironment, characterized by hypoxia and ECM accumulation, influences immune cells, both in promoting inflammation and facilitating the resolution of fibrosis [54]. Various immune cells, such as NK cells, M2 macrophages, Tregs, dendritic cells (DCs), NKT cells, and neutrophils, are essential in both the progression and resolution of fibrosis [55].

NK cells are essential in modulating fibrosis by directly killing activated HSCs, which are the primary collagen‐producing cells in fibrosis. They achieve this through mechanisms, such as the release of perforin and granzymes, as well as through pathways involving tumor necrosis factor‐related apoptosis‐inducing ligand (TRAIL), Fas ligand (FASL), and NKG2D [56]. Additionally, NK cells can modulate the activity of other immune cells, like macrophages, by producing interferon‐gamma (IFN‐γ), which steers macrophage polarization toward an antifibrotic M2 macrophage phenotype [57]. Functionally, cytokines, such as interleukin (IL)‐15, enhance NK cell proliferation and activation, improving fibrosis resolution in experimental models [58]. Monoclonal antibodies promoting NK cell‐mediated antibody‐dependent cellular cytotoxicity (ADCC) have shown efficacy in preclinical studies [59]. Nonetheless, the regulation of NK cell activity in the fibrotic microenvironment, including overcoming HSC‐mediated immunosuppression, remains a critical research focus to optimize NK‐based immunotherapies.

On the other hand, macrophages, including Kupffer cells, have a central role in regulating the resolution of inflammation and fibrosis through phagocytosis of apoptotic HSCs and secretion of MMPs to degrade ECM [60]. This clearance by macrophages not only prevents sustained inflammation, but also promotes a shift from a proinflammatory to a restorative macrophage phenotype, which favors liver tissue recovery [1]. Thus, the synergy between activated HSCs apoptosis and the effector functions of NK cells and macrophages provides the foundation for the resolution of liver fibrosis and restoration of normal liver function.

Tregs play a protective role in liver fibrosis by suppressing excessive inflammatory responses and modulation of HSCs [61]. Mouse models of liver fibrosis induced by BDL or toxic injury show that depletion of Tregs leads to enhanced inflammatory cytokine production (TNF‐α and IL‐17) and increased recruitment of fibrogenic macrophages and effector T cells, worsening fibrosis [62]. IL‐10 released from Tregs promotes M2 macrophage polarization, which further secretes anti‐inflammatory mediators facilitating ECM degradation and fibrosis resolution. This Treg‐macrophage axis presents a critical feedback loop suppressing persistent HSC activation and collagen deposition [62, 63].

Deficiency of autophagy, modeled by loss of essential genes, such as ATG5 in T cells, promotes a pathogenic Th17 phenotype characterized by increased glycolytic metabolism and elevated secretion of type 3 cytokines, including IL‐17A and GM‐CSF. These pathogenic T cells drive fibrogenesis by inducing proinflammatory activation of hepatic myofibroblasts, hepatocytes, and macrophages, thereby exacerbating liver fibrosis in murine models. Importantly, autophagy impairment in CD4 T cells is also observed in patients with advanced liver fibrosis, and restoration of autophagy decreases the frequency of pathogenic Th17 cells and attenuates fibrosis. These findings position autophagy in CD4 T cells as a key immune‐metabolic checkpoint and a promising target for therapeutic intervention aimed at controlling inflammation‐driven liver fibrosis [64].

DCs are crucial in antigen presentation and modulating immune responses by promoting Treg expansion to reduce fibrosis progression. They also produce cytokines that influence the activity of NK cells and macrophages, adding to the complexity of the immune response in the liver [65]. NKT cells, which bridge innate and adaptive immunity, produce various cytokines, including IFN‐γ and IL‐4 which enhances NK cell activity against fibrosis. Neutrophils play a dual role in liver fibrosis; while they are often associated with early inflammatory responses that exacerbate liver injury by releasing reactive oxygen species, they can also contribute to fibrosis resolution by promoting the apoptosis of activated HSCs [9].

Inflammatory mediators, including TNF‐α, IL‐6, CCL2, and TGF‐β are major mediators that promote HSCs activation and fibrosis progression. TNF‐α, produced by macrophages and activated HSCs, activates HSCs and increases collagen production through the NF‐κB pathway, creating a feedback loop that exacerbates inflammation and fibrosis [66]. IL‐6 contributes to HSCs activation and TGF‐β production, which drives fibrogenesis, and activates the STAT3 pathway associated with increased collagen synthesis in HSCs [67]. CCL2 or monocyte chemoattractant protein‐1 (MCP‐1) recruit monocytes and macrophages to the liver during inflammation, further activating HSCs and worsening fibrosis [68]. The intricate relationships among TNF‐α, IL‐6, CCL2, and TGF‐β through positive feedback loops and synergistic interactions create a persistent fibrotic environment, suggesting that a multifaceted therapeutic approach may be necessary to address liver fibrosis effectively.

### 2.3. Crosstalk Between the Immune System and HSCs

**Figure 2:**
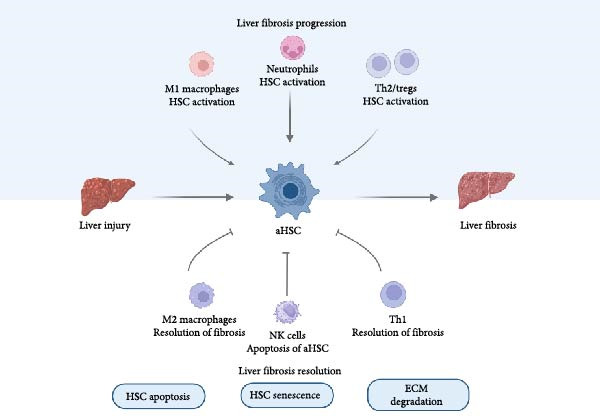
Mechanisms of development and resolution of liver fibrosis. Liver injury induces activation of hepatic stellate cells (activated HSCs), leading to liver fibrosis. M1 macrophages, neutrophils, and Th2/Tregs promote HSCs activation and fibrosis progression. In contrast, M2 macrophages, NK cells, and Th1 cells contribute to the resolution of fibrosis.

HSCs are essential in liver homeostasis, particularly in the context of liver fibrosis and its potential resolution (Figure 2). Activated HSCs generate an immunotolerant microenvironment within fibrotic liver tissue, primarily through the secretion of immunosuppressive cytokines and molecules such as TGF‐β, IL‐6, and programmed death ligand 1 (PD‐L1). TGF‐β is particularly influential as it not only promotes HSCs activation and subsequently collagen production, but also inhibits the proliferation and function of various immune cells, including NK and T helper cells, thus contributing to a state of immune tolerance [69]. IL‐6 further exacerbates this immunosuppressive environment by promoting the differentiation of Tregs, which are known to suppress the activity of effector T cells and NK cells, thus dampening the immune response [62]. In addition, PD‐L1 expression on activated HSCs serves to inhibit T cell activation and proliferation, reinforcing the immunosuppressive environment [70].

Moreover, the recruitment of immune cells to the liver is significantly influenced by chemokines secreted by activated HSCs. For example, activated HSCs produce chemokines such as CCL2, CCL3, and CCL5, which facilitate the recruitment of various immune cell types to the site of injury, thereby shaping the inflammatory milieu within the fibrotic liver [71]. This recruitment is essential for the resolution of inflammation and the potential regression of fibrosis, highlighting the dynamic nature of the immune response in liver pathology [72].

From immune cells, NK cells can recognize and destroy activated HSCs, an essential process for halting fibrosis progression and supporting liver regeneration [73]. Specific macrophage subtypes, such as M2 macrophages, play an anti‐inflammatory role by secreting cytokines like IL‐10 that promote HSCs apoptosis and ECM breakdown, helping to resolve fibrosis [74]. Conversely, M1 macrophages, which are proinflammatory, release cytokines that continue to activate HSCs and increase collagen production, exacerbating the fibrosis [75]. Thus, the balance between profibrotic (M1) and antifibrotic (M2) signals is critical for liver disease outcomes, as a predominance of M1 can worsen fibrosis, whereas M2 dominance can support tissue healing and resolution [74, 76].

T cells, particularly T helpers (Th1 and Th2) and Tregs, contribute significantly to liver disease progression through distinct cytokine profiles. Th1 cells produce IFN‐γ, which support antifibrotic effects in liver fibrosis by suppressing HSC activation, reducing collagen and ECM deposition, and promoting apoptosis or quiescence of activated HSCs. For example, in vivo studies in rat models showed that IFN‐γ treatment reduced α‐smooth muscle actin‐positive HSCs and decreased mRNA levels of procollagen I, fibronectin, and laminin [63, 77]. In contrast, Th2 cells secrete IL‐13 and IL‐4, which strongly promote fibrosis. A recently described IL‐13‐producing intrahepatic ILC3‐like population is enriched in human fibrotic livers and can directly stimulate human HSCs, inducing a proinflammatory and profibrotic transcriptional program. Moreover, IL‐4 and IL‐13 signaling, via IL‐4Rα–STAT6, has been implicated in promoting fibrosis in multiple contexts by increasing ECM production and profibrotic gene expression [63, 78]. Tregs act via immunosuppressive cytokines (IL‐10 and TGF‐β), repressing proinflammatory and Th17‐mediated signals, modulating NK cell and Kupffer cell activity, and directly influencing HSC fate. However, Tregs play a dual role that also contribute to fibrogenesis via accumulating in fibrotic livers and suppressing MMP expression by Kupffer cells, thereby tipping the balance toward ECM accumulation [62, 79]. The balance of Th1, Th2, and Treg cells’ activity is, thus, crucial in the management of liver fibrosis with Th2/Tregs promoting fibrogenesis and Th1 supporting fibrosis resolution.

Neutrophils contribute to HSCs activation through the release of ROS and proinflammatory cytokines such as TNF‐α, IL‐17, IL‐6, and CCL2. ROS, produced through NOX pathways, not only damages hepatocytes but also activates HSCs, encouraging their transition to a myofibroblast phenotype associated with fibrogenesis. This oxidative stress triggers profibrogenic signaling pathways, increasing collagen production and promoting fibrosis progression [80].

Recent research has also emphasized on signaling pathways and interactions between HSCs and immune cells, contributing to liver fibrosis development. For instance, single‐cell transcriptomic profiling studies reveal that interactions between HSCs and immune cells, such as macrophages and T cells, can activate HSCs through the secretion of proinflammatory cytokines, like TNF and IL‐1 family, exacerbating fibrogenesis [81]. Furthermore, exosomes derived from HSCs and immune cells facilitate intercellular communication, and modulate both fibrogenic responses and resolution mechanisms [82]. Summary of immune cell involvement in liver fibrosis and their crosstalk with HSCs are presented in Table 1.

**Table 1:** Immune cell contributions to liver fibrosis and crosstalk with HSCs.

Immune cell type	Profibrotic role	Antifibrotic role	Key cytokines/mechanisms	Signaling pathways
Natural killer (NK) cells	Rare profibrotic role, sometimes via cytokine imbalance	Induce apoptosis of activated HSCs, promote fibrosis resolution	IFN‐γ, TRAIL	TRAIL, perforin/granzyme pathways

M1 macrophages	Promote inflammation, activate HSCs, produce ROS and proinflammatory cytokines	Limited antifibrotic effects	TNF‐α, IL‐1β, IL‐6, ROS	NF‐κB, STAT3

M2 macrophages	Facilitate fibrosis via macrophage‐to‐myofibroblast transition (MMT), secretion of profibrotic mediators such as TGF‐β and arginase‐1	Promote fibrosis resolution, secrete anti‐inflammatory cytokines, and degrade ECM	IL‐10, TGF‐β	TGF‐β/Smad3, IL‐10 signaling

Regulatory T cells (Tregs)	Potential suppression of immune surveillance if dysregulated	Suppress inflammation and HSC activation, promote M2 macrophage polarization	IL‐10, TGF‐β	IL‐10, TGF‐β signaling

Dendritic cells (DCs)	Can stimulate fibrogenic immune responses	Modulate immune tolerance and fibrosis resolution	IL‐12, IL‐10	Various cytokine pathways

Natural killer T (NKT) cells	Promote fibrosis via secretion of IL‐4, IL‐13	Can enhance NK cell antifibrotic activity	IFN‐γ, IL‐4, IL‐13	Multiple

Neutrophils	Release ROS and proinflammatory cytokines, activate HSCs	Can induce apoptosis of activated HSCs	TNF‐α, IL‐6, IL‐17, ROS	NF‐κB, ROS pathways

Th1 cells	Can have antifibrotic effects via IFN‐γ production	–	IFN‐γ	STAT1

Th2 cells	Promote fibrosis through profibrotic cytokines	–	IL‐4, IL‐13	STAT6

Th17 cells	Promote fibrosis via IL‐17 production, HSC activation	—	IL‐17, IL‐22	ERK1/2, p38, STAT3

## 3. Therapeutic Potential of Targeting Immune System and HSCs Crosstalk

### 3.1. Current Therapies for Liver Fibrosis

Current therapies for liver fibrosis primarily focus on addressing the underlying causes, such as viral hepatitis or alcohol consumption, but they often fall short of effectively reversing established fibrosis. The limitations of these therapies highlight the need for innovative approaches that target the intricate crosstalk between the immune cells and HSCs. One of the primary challenges in treating liver fibrosis is the modulation of HSCs activity. This process is exacerbated by inflammatory mediators from the immune cells, such as neutrophils and macrophages, which activate HSCs through various signaling pathways [83]. These interactions complicate the therapeutic landscape, as targeting HSCs alone may not suffice without addressing the immune microenvironment that sustains their activation.

Existing therapies, such as antiviral treatments for hepatitis C or corticosteroids for autoimmune hepatitis, do not directly target the fibrotic process itself and may have limited efficacy in advanced fibrosis. Antifibrotic agents, like Imatinib, a Bcr‐Abl inhibitor and a blocker of various tyrosine kinases, is currently under evaluation in individuals with liver fibrosis, despite its previous failure in treating idiopathic pulmonary fibrosis (IPF). Additionally, dasatinib is undergoing trials for its efficacy in liver fibrosis [84]. While antifibrotic agents, like pirfenidone and nintedanib, which inhibit fibrogenic signaling pathways, have shown promise in preclinical models and clinical trials, their effectiveness in reversing fibrosis remains uncertain, and they often come with significant side effects [85]. Recently, resmetirom, a thyroid hormone receptor β‐selective agonist, has demonstrated promising results in reducing liver fat and improving fibrosis biomarkers in nonalcoholic steatohepatitis (NASH), positioning it as a potential therapeutic for liver fibrosis management [86]. Moreover, the heterogeneity of liver diseases complicates the development of one‐size‐fits‐all treatments, necessitating a more personalized approach that considers the specific immune–HSCs interactions at play in each patient.

Recently, therapeutic interventions that target the interaction between the immune system and HSCs are emerging as promising strategies in the management of liver fibrosis. One innovative approach is immunotherapy that targets the immune system cells/components to modulate the chronic inflammatory processes driving fibrosis progression. This therapy aims to modulate/regulate the immune responses to halt or even reverse fibrosis [87]. In addition, exosome‐based therapies are being explored to alter the fibrogenic pathway in HSCs. These exosomes may deliver therapeutic molecules capable of modulating HSCs behavior, potentially restoring HSCs from their activated state and reducing fibrosis [88]. Notably, exosomes derived from mesenchymal stromal cells (MSCs) have been shown to carry several antifibrotic miRNAs such as miR‐133a, miR‐29b, miR‐146a, miR‐181‐5p, and miR‐223 [89–93]. These miRNAs inhibit HSC activation and suppress fibrogenic signaling pathways. For instance, miR‐133a, delivered via Wharton’s jelly MSC‐derived exosomes, targets the TGF‐β signaling/Smad3 pathway to reduce ECM deposition and mitigate fibrogenesis by reduced expression of profibrotic markers and upregulation of miR‐133a after exosome treatment [91]. Similarly, miR‐29b exerts antifibrotic roles by downregulating collagen synthesis and ECM remodeling factors [94]. Engineered exosomes enriched in miR‐181‐5p also attenuate fibrosis by activating autophagy and downregulating STAT3/Bcl‐2 pathways in HSCs [90]. Additionally, exosomes carry proteins and other regulatory molecules that modulate immune responses and promote fibrosis resolution. These molecular cargos represent promising therapeutic agents by modulating HSC proliferation, inflammatory responses, and ECM remodeling, thereby supporting the therapeutic potential of exosome‐based interventions in liver fibrosis [92]. While this approach is promising, additional evidence from the literature is needed to support these specific claims.

Furthermore, the use of certain microRNAs such as miR‐185 is also of interest as antifibrotic agents. These microRNAs work by inhibiting HSCs activation and promoting apoptosis through downregulation of profibrotic signaling pathways. Clinical trials regarding the delivery of this microRNA are ongoing, demonstrating its potential as a novel therapeutic agent for managing liver fibrosis [95]. Taken together, these innovative strategies highlight the importance of targeting immune‐HSCs interactions as a promising multifaceted approach to reversing liver fibrosis and restoring liver function.

### 3.2. Modulating Immune Responses to Control HSCs Activation

A variety of strategies have been explored to inhibit HSCs activation through immune modulation, particularly focusing on the roles of proinflammatory cytokines such as TGF‐β and IL‐6 [96]. Inhibiting TGF‐β signaling has emerged as a potential therapeutic target. For instance, asiatic acid has been shown to attenuate CCl4‐induced liver fibrosis in rats and suppress TGF‐β1‐stimulated activation of HSCs in vitro by upregulating Smad7, thereby blocking Smad2/3 phosphorylation, reducing α‐SMA and collagen matrix expression [97]. Similarly, knockdown of TGF‐β1 via siRNA in rat models reduces type I and III collagen synthesis and diminishes histological fibrosis [98]. Additionally, the use of small molecule inhibitors that target the TGF‐β receptor or downstream signaling pathways has shown promise in preclinical models, suggesting that these strategies could be translated into clinical applications [99].

IL‐6 is another critical cytokine involved in HSCs activation and liver fibrosis. It has been shown to enhance the fibrogenic activity of HSCs by promoting their proliferation and survival [100]. Targeting IL‐6 signaling pathways, either through monoclonal antibodies or small molecule inhibitors, has been proposed as a strategy to inhibit HSCs activation. For example, blocking IL‐6 via disrupting the HLF/IL‐6/STAT3 circuit has been associated with reduced HSCs induction and improved liver function in experimental models of liver fibrosis [101]. Similarly, Imatinib has been demonstrated to suppress HSC activation via targeting the IL‐6/STAT3 pathway in vitro and in vivo [102].

In addition to targeting specific cytokines, modulating the immune cell populations that interact with HSCs can influence HSCs activation. Therapies aimed at boosting overall immune surveillance in the liver fibrosis can be achieved through the modulation of the liver microenvironment to reduce the immunosuppressive effects of HSCs. For instance, Tregs have been shown to exert immunosuppressive effects that can inhibit HSCs activation and promote fibrosis resolution [103]. Enhancing Treg activity through the manipulation of HSCs or their microenvironment may provide a therapeutic avenue to control HSCs modulation. Moreover, the role of myeloid‐derived suppressor cells (MDSCs) in the liver has been recognized as a potential mechanism through which HSCs can modulate immune responses and promote tolerance, further complicating the immune landscape in liver fibrosis [104]. Modulating the signaling pathways that regulate the activation and function of these immunosuppressive cells could help re‐establish a more robust immune response against activated HSCs and strengthen the antifibrotic effect.

Furthermore, inhibitors of NF‐κB have been shown to promote the resolution of fibrosis by affecting HSCs viability and modulating the secretion of various chemokines and cytokines [105]. This suggests that targeting NF‐κB signaling could represent a viable strategy for inhibiting HSCs activation and promoting liver repair.

Referring to the central role of the PDGF signaling pathway in HSCs activation and liver fibrosis, therapeutic approaches targeting this pathway have great potential. PDGFR blockers have shown promising effects in preclinical models to induce HSCs modulation and prevent fibrosis progression [25]. For example, neutralizing antibodies against PDGF‐BB or small molecule inhibitors targeting PDGFR‐β have been shown to reduce liver fibrosis in experimental models, indicating their potential application in clinical medicine [26]. In addition to inhibiting HSCs proliferation, therapies that modulate the PDGF signaling pathway may promote apoptosis or return of HSCs to a quiescent state, which favors liver tissue regeneration and prevents fibrosis progression [106].

### 3.3. Macrophage Polarization Therapies

Macrophage polarization therapies have emerged as a critical area of research in the management of fibrosis, particularly in the liver and kidneys. Macrophages can be polarized into two main phenotypes: the proinflammatory M1 macrophages and the anti‐inflammatory M1 macrophages promote inflammation and HSC activation via TNF‐α, IL‐1β, and ROS production, activating NF‐κB and STAT3 pathways that exacerbate fibrosis [76]. M2 macrophages. M2 macrophages are well‐known for their anti‐inflammatory properties, primarily through the secretion of cytokines, such as IL‐10, IL‐4, and TGF‐β, which facilitate tissue repair and the resolution of fibrosis. However, M2 macrophages can also play a role in the advancement of fibrosis by producing profibrotic factors like TGF‐β and arginase‐1, which activate HSCs and promote the deposition of ECM [74, 107]. The IL‐4/IL‐13/STAT6 signaling pathway serves as a key mechanism that drives M2 polarization towards a reparative phenotype, whereas persistent activation of TGF‐β/Smad signaling in M2 macrophages can enhance fibrogenesis [74, 108]. This dual functionality highlights the complexity in therapeutically targeting M2 macrophages, necessitating strategies that carefully enhance their tissue repair functions without worsening fibrosis. Ongoing research is vital to develop selective modulation approaches that capitalize on the advantageous properties of M2 macrophages while reducing their fibrogenic effects [76, 108]. Therefore, strategies aimed at promoting M2 macrophage activity are being explored as potential therapeutic interventions to mitigate fibrosis and enhance tissue regeneration [109].

One of the primary approaches to promote M2 macrophage activity involves the manipulation of cytokine signaling pathways. IL‐4 and IL‐13 are key cytokines that drive the polarization of macrophages towards the M2 phenotype. The IL‐4 receptor alpha (IL‐4Rα) plays a central role in this process, and its activation leads to the expression of various genes associated with M2 macrophage functions, including MMPs that facilitate tissue remodeling [110].

Another promising avenue for promoting M2 macrophage activity involves the use of pharmacological agents that can enhance macrophage polarization. For example, certain drugs, such as pioglitazone, have been shown to increase the expression of vascular endothelial growth factor receptor‐3 (VEGFR3) and promote M2 macrophage activation through the peroxisome proliferator‐activated receptor gamma (PPAR‐γ) pathway [111]. This suggests that pharmacological modulation of macrophage polarization could be a viable therapeutic strategy for managing fibrosis. Additionally, compounds that inhibit M1 polarization or promote M2 polarization, such as hydroxychloroquine, have demonstrated efficacy in reducing fibrosis in various models by selectively modulating macrophage activity [112].

The role of macrophage‐to‐myofibroblast transition (MMT) is also crucial in the context of fibrosis. M2 macrophages can contribute to the activation of fibroblasts and the production of ECM components, which are hallmarks of fibrotic tissue [113]. Therefore, targeting signaling pathways such as TGF‐β/Smad3, which are implicated in MMT, may help to prevent the fibrogenic effects of M2 macrophages and promote a more balanced tissue repair response [114].

### 3.4. NK Cell‐Based Therapies

NK cell‐based therapies have gained significant attention as a potential strategy for enhancing immune responses against activated HSCs in liver fibrosis [115]. One of the primary approaches involves the use of cytokines and growth factors that can stimulate NK cell proliferation and cytotoxicity. IFN‐γ is particularly noteworthy, as it not only enhances the cytotoxic functions of NK cells but also promotes the expression of activating ligands on target cells, including HSCs [116]. Studies have shown that treatment with IFN‐γ can significantly increase the ability of NK cells to recognize and kill activated HSCs, thereby reducing fibrogenesis in experimental models of liver injury [117]. Additionally, other cytokines such as IL‐15 have been explored for their ability to expand NK cell populations and enhance their functional capabilities, further supporting their role in targeting activated HSCs [103].

Another promising strategy involves the use of monoclonal antibodies or antibody‐based therapies that can enhance NK cell‐mediated cytotoxicity against activated HSCs. For instance, antibodies targeting specific surface markers on activated HSCs can facilitate ADCC, a mechanism through which NK cells are recruited to eliminate target cells [118]. This approach not only enhances the direct cytotoxic effects of NK cells but also helps to modulate the immune microenvironment in the liver, potentially reversing the immunosuppressive effects often associated with chronic liver diseases [119].

Moreover, the recruitment of NK cells to the liver can be augmented by manipulating chemokine signaling pathways. Activated HSCs express various chemokines that can attract NK cells, and enhancing the expression of these chemokines may improve NK cell infiltration into fibrotic liver tissue [120]. For example, the chemokine CCL5 has been shown to play a role in recruiting NK cells to sites of inflammation, and its upregulation in the liver could be a strategic target for enhancing NK cell responses against activated HSCs [121].

Furthermore, the use of small molecules that inhibit immunosuppressive pathways, such as indoleamine 2,3‐dioxygenase (IDO) or PD‐1 signaling, can also enhance NK cell activity and promote immune surveillance [122]. These inhibitors can help to reverse the immunosuppressive microenvironment created by activated HSCs, thereby allowing NK cells to exert their cytotoxic effects more effectively.

## 4. Emerging Research and Therapeutic Directions

Recent studies have shed light on the role of specific immune cell subsets in the progression of liver fibrosis. For instance, mucosal‐associated invariant T (MAIT) cells have been identified as profibrogenic, promoting HSCs activation and ECM deposition through the secretion of profibrogenic cytokines [123]. Similarly, innate lymphoid cells have been implicated in the fibrogenic process, particularly in the context of chronic liver injury. Understanding these interactions provides insights into potential therapeutic targets for liver fibrosis, where modulation of immune responses could lead to improved outcomes in patients with chronic liver diseases [124].

Innovative strategies to tackle liver fibrosis also emphasize cellular therapy to treat chronic liver diseases, including cirrhosis and liver failure. Stem cell therapy, such as the use of MSCs, shows a promising potential in alleviating fibrosis by modulating activated HSCs and promoting liver tissue regeneration [125]. Studies confirm that MSC therapy can significantly improve liver function and reduce fibrotic tissue, particularly by suppressing profibrotic pathways such as TGF‐β and deactivating HSCs [126, 127].

Deeper insights into the liver fibrosis mechanisms, including the roles of mediators like exosomal miRNAs, pave the way for more effective therapies for liver fibrosis [10, 128]. Exosomes loaded with specific microRNAs or regulatory proteins can regulate the activation of HSCs and modulate immune responses, offering targeted delivery and the potential for reduced off‐target effects. For example, exosome‐mediated delivery of antifibrotic miRNAs has been shown to modulate HSCs activation and attenuate fibrosis in preclinical models. Zhang et al. [93] demonstrated that engineered exosomes efficiently delivered miR‐29b to activated HSCs, suppressed their activation by downregulating fibrotic markers, such as α‐SMA and COL1A1, and significantly reduced collagen deposition and fibrosis in liver fibrosis models by inhibiting the TGF‐β/Smad signaling pathway. He et al. [129] demonstrated that exosomal miR‐223 originated from neutrophils, inhibits the expression of HSC activation markers and reduces fibrosis progression through repression of profibrotic genes and inflammatory mediators. Similarly, Boonkaew et al. [130] reported that miR‐223‐containing exosomes reduce lipid accumulation, inflammation, and fibrotic marker expression in liver cells, emphasizing its therapeutic potential in liver diseases. These exosomes rich in miR‐223 are selectively internalized by hepatocytes via low‐density lipoprotein receptors (LDLRs), facilitating the suppression of profibrotic genes, such as *TAZ* (transcriptional co‐activator with PDZ‐binding motif) and inflammatory cytokines, resulting in reduced steatosis and fibrosis. Moreover, IL‐6 signaling in myeloid cells enhances the packaging of miR‐223 into exosomes, supporting the crosstalk between immune cells and hepatocytes to resolve liver injury [129, 130].

Moreover, stem cell‐derived exosomes, secreted by MSCs, can modulate liver inflammation, fibrosis, and regeneration. Preclinical studies have shown that these exosomes can attenuate liver fibrosis by inhibiting activation of HSCs, regulating macrophage polarization, and suppressing profibrotic signaling pathways like Hedgehog/SMO and STAT3. For example, the exosomes enriched with miR‐148a, reduced inflammation and fibrosis by targeting the KLF6/STAT3 pathway macrophages [131, 132].

Recent mechanistic studies identify hypoxia‐driven stabilization of HIF‐1α and Wnt/β‐catenin activation as central barriers to immune infiltration and immunotherapy success. Combination preclinical studies, using immune checkpoint inhibitors (ICIs) with anti‐VEGF or local ablation, demonstrate increased T‐cell recruitment and HSC modulation, decreasing immunosuppression in the tumor stroma. Additionally, the use of chimeric antigen receptor (CAR)‐T cells engineered against GPC3 or other liver cancer antigens is showing enhanced cytolytic activity in fibrotic and tumorous livers, though on‐target/off‐tumor effects remain a challenge [133, 134]. Collectively, these findings indicate how mechanistic deconvolution of immune resistance yields rational combinations to overcome microenvironmental barriers in liver disease.

## 5. Opportunities for Combining Immunotherapies With Antifibrotic Agents

The crosstalk between the immune system and HSCs presents a promising avenue for developing innovative therapies for liver fibrosis. Recent research has highlighted the potential for combining immunotherapies with antifibrotic agents to enhance therapeutic outcomes in liver fibrosis [135, 136]. One of the key strategies involves targeting the inflammatory pathways that contribute to HSCs activation. For instance, the modulation of immune responses through the use of monoclonal antibodies, such as those targeting claudin‐1, has shown promise in inhibiting fibrosis by altering cell plasticity and reducing the fibrogenic potential of HSCs. This approach not only addresses the fibrotic process but also has implications for preventing HCC, which often arises in the context of advanced liver fibrosis [137].

Moreover, the use of antioxidants has emerged as a viable antifibrotic strategy. Antioxidants can mitigate oxidative stress, a significant contributor to HSCs activation and subsequent fibrosis progression. For example, L‐histidine has been identified as an antioxidant that can attenuate liver fibrosis by inhibiting collagen synthesis and reducing oxidative stress [138]. This suggests that combining antioxidant therapies with immunomodulatory treatments could provide a dual benefit by addressing both the inflammatory and fibrotic components of liver disease.

In addition to antioxidants, the modulation of energy metabolism in HSCs has been explored as a potential antifibrotic strategy. Research indicates that targeting the metabolic pathways in activated HSCs can reverse their fibrogenic phenotype without adversely affecting hepatocyte function [139]. This approach could be synergistic with immunotherapies that aim to reduce inflammation, thereby creating a more favorable environment for liver regeneration.

Furthermore, gene therapy targeting MMPs has shown promise in preclinical models. For instance, liver‐targeted hydrodynamic gene delivery of MMP‐13 has been effective in preventing liver fibrosis by promoting ECM degradation and inhibiting HSCs activation [140]. This strategy could be combined with immunotherapies that enhance the immune response against activated HSCs, potentially leading to a more robust antifibrotic effect.

Recent mechanistic evidence demonstrates that targeting key transcription factors involved in HSC activation offers a promising antifibrotic strategy. For instance, Solhi et al. [141] employed a novel XBP1‐specific decoy oligodeoxynucleotide to directly modulate activated HSCs, resulting in significant downregulation of fibrogenic markers such as α‐SMA, LOX, and TIMP1, as well as reduced collagen secretion and impaired HSC migration. This primary research provides critical experimental validation for modulating ER stress‐related pathways in fibrogenesis [141].

Combining stem cell therapies with conventional antifibrotic treatments offers a new and innovative strategy. Studies have demonstrated that stem cell‐conditioned media can exert antifibrotic effects, and when combined with certain agents like nilotinib, a tyrosine kinase inhibitor, the therapeutic efficacy is significantly enhanced. The co‐administration improved liver function, reduced oxidative stress, apoptosis, and collagen deposition in CCL4‐induced liver fibrosis in rats, outperforming monotherapies in attenuating fibrosis [142]. Similarly, in a rat model of liver fibrosis, co‐treatment with bone marrow‐derived MSCs and Imatinib, another tyrosine kinase inhibitor, showed enhanced reduction of fibrotic markers, such as α‐SMA and collagen, with better histopathological improvement than either therapy alone [143]. A meta‐analysis of preclinical studies supports that MSCs combined with various drugs can more effectively repair damaged liver tissue and regulate key fibrotic signaling pathways, leading to improved antifibrotic effects over MSC monotherapy [127].

In summary, the combination of immunotherapies with antifibrotic strategies, including antioxidants, metabolic modulators, gene therapies, and stem cell‐based methods, shows potential for developing more effective treatments. Further investigation into the mechanisms driving these interactions and refining combination therapies will be essential for enhancing outcomes in patients with chronic liver diseases.

## 6. Future and Challenges in Targeting Immune System and HSCs Crosstalk

The intricate interplay between immune cells and HSCs is central to the pathogenesis of liver fibrosis. Understanding this crosstalk presents both opportunities and challenges for therapeutic interventions aimed at modulating immune responses and fibrosis stages. The heterogeneity of immune responses and the dynamic nature of fibrosis progression necessitate a nuanced approach to targeting these interactions.

Immune cells, like NK cells, M2 macrophages, Tregs, DCs, and NKT cells, perform various functions that can either contribute to or alleviate fibrosis, depending on the situation. For instance, while NK cells can directly kill activated HSCs and promote fibrosis resolution [56], M2 macrophages can contribute to fibrogenesis through the secretion of profibrotic factors. The balance between these opposing roles is influenced by the stage of fibrosis and the local inflammatory milieu, which is characterized by cytokines such as TNF‐α, IL‐6, CCL2, and TGF‐β [123].

The progression of liver fibrosis is not linear; it involves phases of inflammation, activation, and resolution. During early stages, immune cells may work collaboratively to initiate repair processes, while in advanced fibrosis, the same cells may exacerbate the fibrotic response [144]. This dynamic necessitates a thorough understanding of the timing and context of immune interventions, as targeting immune cells at the wrong stage could lead to unwanted immune suppression or exacerbation of fibrosis [62, 145].

Targeting immune responses to promote fibrosis resolution carries the risk of unintended immune suppression. For example, enhancing Treg activity to mitigate inflammation may inadvertently dampen the immune response to infections or malignancies [146]. Similarly, while promoting M2 macrophage polarization can aid in tissue repair, excessive M2 activation may lead to increased collagen deposition and fibrosis [147]. Therefore, careful modulation of immune responses is required to avoid compromising host defenses.

Conversely, interventions aimed at enhancing proinflammatory responses may exacerbate fibrosis. For instance, strategies that boost NK cell activity could lead to increased inflammation if not properly regulated, potentially resulting in further HSCs activation and collagen deposition [56]. Moreover, the use of cytokines, such as IL‐6, to stimulate immune responses must be approached with caution, as elevated IL‐6 levels are associated with increased fibrosis in various liver diseases [123].

Identifying biomarkers that reflect the immune status and fibrosis stage could facilitate personalized treatment strategies. For example, profiling immune cell populations and cytokine levels in patients could help tailor interventions to the specific needs of individuals based on their disease stage and immune profile [144, 148].

Further elucidation of the mechanisms underlying immune system and HSCs crosstalk is essential for developing effective therapies. Investigating how different immune cells interact with HSCs and the signaling pathways involved will provide insights into potential therapeutic targets [56, 123]. For example, understanding the role of specific cytokines in mediating these interactions could lead to the development of novel inhibitors that selectively disrupt fibrogenic signaling pathways. Moreover, the ability of stem cell‐derived extracellular vesicles to reprogram immune cell function represents a significant advancement in antifibrotic therapy. The study by Torabi et al. [149] provides compelling preclinical evidence that Wharton’s Jelly‐MSC‐derived EVs can shift macrophages toward a phenotype that supports fibrosis resolution, offering a novel, cell‐free approach for future clinical development.

The future of targeting immune system and HSCs crosstalk in liver fibrosis holds great promise but is fraught with challenges. The heterogeneity of immune responses and the dynamic nature of fibrosis progression necessitate a careful and informed approach to therapeutic interventions. Focusing on targeted therapies, personalized approaches, and a better understanding of immune mechanisms could enable effective modulation of immune responses and reduce liver fibrosis, without the risks of unwanted immune suppression or worsening of the condition.

## 7. Conclusion

The interplay between immune cells and HSCs is central to the pathogenesis of liver fibrosis. HSCs activation, driven by immune‐mediated signaling pathways and inflammatory cytokines, plays a pivotal role in ECM deposition and fibrogenesis. Important immune cells, such as macrophages, NK cells, and Tregs, play dual roles in either driving or resolving fibrogenesis. These dynamic interactions underscore the significance of immune system and HSCs crosstalk in fibrosis progression and its potential resolution, particularly in the early stages of liver injury.

Further investigation into the molecular mechanisms governing immune system and HSCs interactions is crucial for developing more targeted and effective therapies for liver fibrosis. Strategies, such as modulating macrophage polarization, enhancing NK cell activity, and targeting cytokine signaling pathways, including TGF‐β and IL‐17, hold promise for mitigating fibrosis. Additionally, the integration of immunotherapies with antifibrotic agents may offer synergistic effects, potentially reversing fibrosis in its early stages. Personalized medicine holds great potential, using breakthroughs in genomics, transcriptomics, and single‐cell technologies to customize treatments for patients according to their distinct immune and fibrotic profiles. This approach could significantly improve clinical outcomes, addressing the current challenges in fibrosis management and paving the way for precision‐based interventions in chronic liver diseases.

**Nomenclature**
ECM: Extracellular matrix
HSCs: Hepatic stellate cells
α‐SMA: Alpha‐smooth muscle actin
MMPs: Matrix metalloproteinases
TGF‐β: Transforming growth factor‐beta
PDGF: Platelet‐derived growth factor
NF‐κB: Nuclear factor‐kappa B
NK cells: Natural killer
NKT cells: Natural killer T
Tregs: Regulatory T cells
TRAIL: Tumor necrosis factor‐related apoptosis‐inducing ligand
MASLD: Metabolic dysfunction‐associated steatotic liver disease.

## Disclosure

All the authors have reviewed and approved the article.

## Conflicts of Interest

The authors declare no conflicts of interest.

## Author Contributions

**Wahyu Widowati and Massoud Vosough:** conceptualization, writing, original draft supervision, validation. **Adilah Hafizha Nur Sabrina** **, Annisa Firdaus Sutendi, Fadhilah Haifa Zahiroh, and Aris Muhamad Nurjamil:** literature search, data curation, writing review. **Teresa Liliana Wargasetia** **, Ita Margaretha Nainggolan, Rizal Azis, and Elham Rismani:** review, editing, validation.

## Funding

The authors received no specific funding for this work.

## Data Availability

Data sharing is not applicable to this article as no datasets were generated or analyzed during the current study.
